# Supplementary material for: Geometry-controlled phase transition in vibrated granular media
Source: Sci Rep. 2022 Sep 2;12:14989. doi: 10.1038/s41598-022-18965-4 (PMC9440227; doi:10.1038/s41598-022-18965-4)
Supplement: Supplementary file 1 — Supplementary Information. [file 41598_2022_18965_MOESM1_ESM.pdf]

# Geometry-controlled phase transition in vibrated granular media (Supplementary Information)

**René Zuñiga[1,2], Germán Varas[2], and Stéphane Job[1,*]**

[1]Laboratoire Quartz, EA-7393, ISAE-Supméca, 3 rue Fernand Hainaut 93400 Saint-Ouen-sur-Seine, France.
[2]Instituto de Física, Pontificia Universidad Católica de Valparaíso, Avenida Brasil 2950, Valparaíso, Chile.
[*]Corresponding author: stephane.job@isae-supmeca.fr

## ABSTRACT

Here we further extend the experimental results, focusing on the dynamics of the variables, studying their variations with the dimensionless acceleration Γ, and present the conditions to define the crystal (solid-like) state. The results follow the same order as in the manuscript, divided into six sections: (i) topological defects, (ii) order parameters, (iii) phase-detection & evolution, and (iv) statistical analysis of the thermal fluctuations. Finally, and in the spirit of having a visual guide of the analyzed quantities, we present animations with the *on-site analysis* of the particles in the experimental setup in (v), before quantifying the particles' mobility in (vi).

## 1 Topological defects

We analyze the temporal evolution of the normalized number of topological defects (disclination and dislocation). First, we consider the disclinations, represented by the particles with $Z = 5$ and $Z = 7$ of nearest neighbors (NN), where $Z$ is the coordination number. These topological defects are always created in pairs; thus, adding these two quantities $(n_5 + n_7)$ gives the total number of disclinations present in the system. We also considered the dislocations $(n_{5\cup 7})$ formed when a bound pair of numbers $Z = 5 \cup 7$ is formed. Figure S1 shows the temporal evolution of both quantities for two conditions, $(\Gamma, \theta) = (10, 10°)$ and $(\Gamma, \theta) = (4, 30°)$. We observed that, in general, the topological defects present oscillations linked to the oscillation of the cell (represented by the soft gray vertical lines). However, their magnitude remains small compared to the average. Comparing both cases, we see a difference in the number of disclinations and dislocations, indicating that the system is closer to a liquid-like state with more defects [Fig. S1 (a)], or to a crystal-like state with fewer defects [Fig. S1 (b)]. We also calculate the temporal average for the number of defects over the entire range of acceleration Γ and angles θ. Figure S1(c) summarized the dislocations

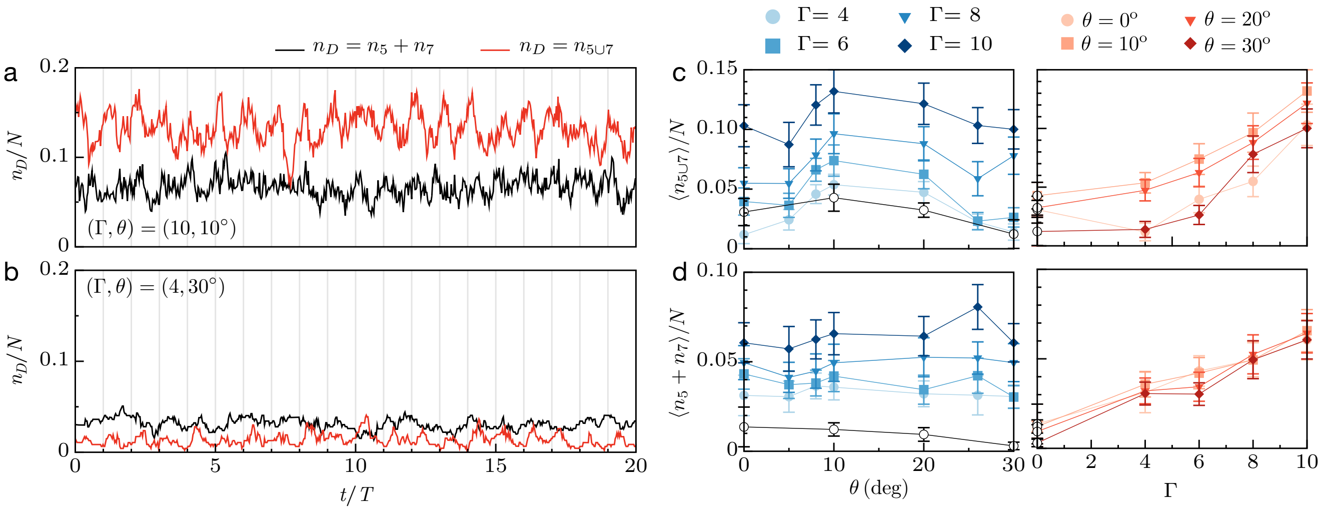


**Figure S1.** Normalized number of topological defects for (a) $(\Gamma, \theta) = (10, 10°)$ and (b) $(\Gamma, \theta) = (4, 30°)$. The black line represent the number of disclinations $(n_5 + n_7)$, and the red lines the total number of dislocations $(n_{5\cup 7})$. (c) Average dislocation as a function of (left) angle θ and (right) acceleration Γ. (d) Average disclination as a function of (left) angle θ and (right) acceleration Γ. The open symbols in (c,d) correspond to the static case, Γ = 0.

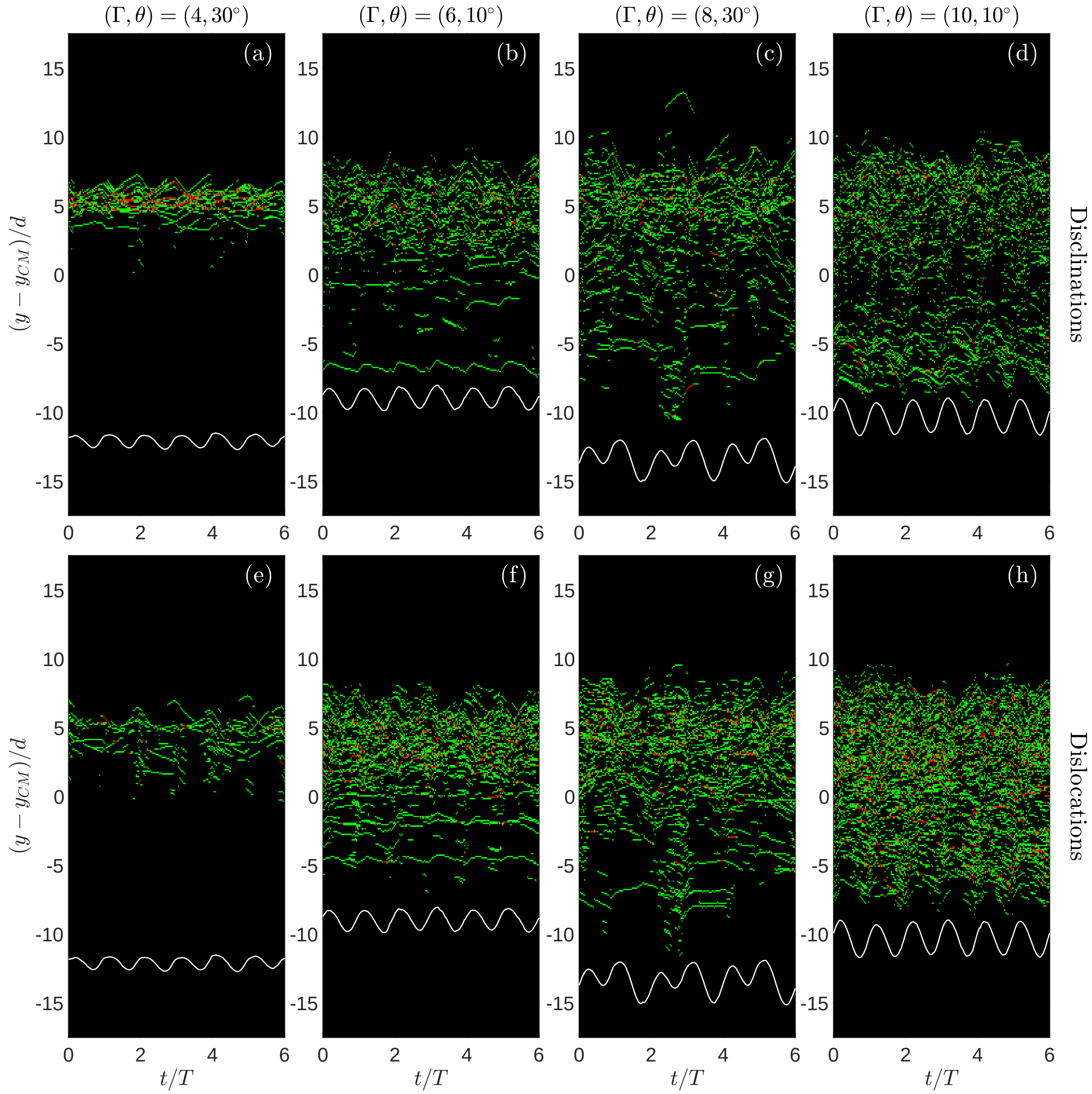


**Figure S2.** Defect's dynamics: spatio-temporal maps of topological defects, versus time $t$ and height $\tilde{y} = y - y_{CM}(t)$ obtained from an instantaneous histogram, counting the defects along the vertical direction, in the frame of the center of mass of all particles. A green (resp. red) dot reveals the presence of a single (resp. two or more) topological defect(s) at a given $(t, \tilde{y})$: (upper row) disclinations and (lower row) dislocations for (columns) the same four examples described in Fig. S6. The white curve indicates the instantaneous position of the apex of the container's V-shape wall in the frame of the center of mass of all particles.

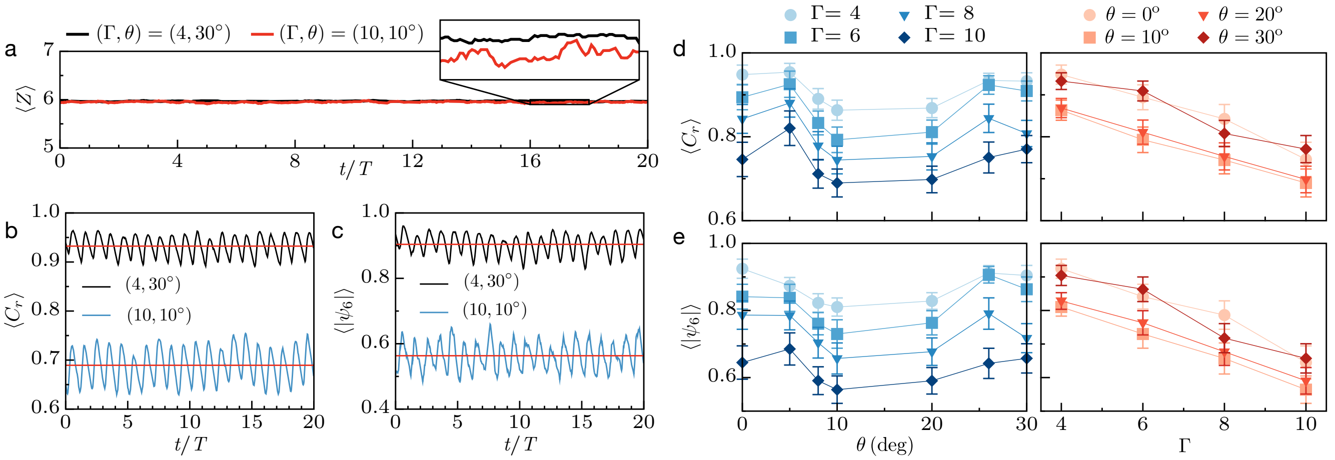


**Figure S3.** (a) Spatial average of the coordination number $\langle Z \rangle$ as a function of time. (b) Average relative compaction $\langle C_r \rangle$ for (black) $(\Gamma, \theta) = (4, 30°)$ and (blue) $(\theta, \Gamma) = (10, 10°)$. The red lines shows the average of the signal. (c) Average local order parameter $\langle |\psi_6| \rangle$ for the same two cases. (d) Average relative compaction as a function of (left) angle $\theta$ and (right) acceleration $\Gamma$. (e) Average order parameter as a function of (left) angle $\theta$ and (right) acceleration $\Gamma$. The error bars correspond to the standard deviation for all experiments.

as a function of the (left) angle and (right) acceleration. The results show a minimum for $\theta = 0°$ and $\theta = 30°$ where they match the symmetry of a hexagonal lattice. Similarly, Figure S1(d) shows the average of the number of disclinations normalized by the total number of particles as a function of the (left) angle and (right) acceleration. As expected, we observe that the number of defects (disclinations and dislocations) increases with increasing vibration energy, i.e., increasing the temperature of the system. In particular, disclinations and dislocations are both detected in the static configuration, see Figure S1(c,d) at $\Gamma = 0$. The activation of these defects, arising from the geometrical asymmetry of the container during the rain-like preparation of the samples, evidences the presence of a liquid-like phase coexisting with the solid-like phase at the initial stage, thus suggesting a phase transition occurring at $kT = 0$. In more details, the combined effects of both the container's geometry and the excitation's amplitude on topological defects is shown in Fig. S2. Such spatio-temporal histograms help revealing the history and evolution of topological defects, here represented as green dots. For instance, a green continuous and horizontal line would represent a permanent and stationary defect, in the frame of the center of mass of the lattice. Instead, a sinuous trajectory reveals a defect traveling (gliding) through the lattice, whereas an intermittent curve reveals its instantaneous creation-annihilation. These mechanisms are specific of how lattices relax to accommodate disorder[1]: interestingly, they are both observable in our experiments, see Fig. S2. In particular, the creation-annihilation is also evidenced by the fluctuations of the defects fraction as a function of time, see Fig. S1 (a).

## 2 Order parameters

We repeat the calculation for the same set of parameters in $(\Gamma, \theta)$ for the relative compaction $C_r$, and order parameter $|\psi_6|$. We observe that the system spends most of its time in a hexagonal array ($Z = 6$) independent of the base angle and vibration amplitude [Fig. S3 (a)]. The average of the coordination number is constant with small fluctuations indicating that the particles maintain a hexagonal arrangement; this leads us to use $|\psi_6|$ to describe the crystal-like state of the system (see Sec. 3). On the other hand, we see a noticeable difference between the two sets of parameters in the relative compaction and order parameter [Fig. S3 (b-c)]. They show a constant average over time and present oscillations linked to the cell vibrating frequency. We observed that for $(\Gamma, \theta) = (4, 30°)$ both quantities are larger than the case $(\Gamma, \theta) = (10, 10°)$. This result shows greater fluidization (smaller value of $C_r$ and $|\psi_6|$) when an angle of asymmetry or higher acceleration occurs. Additionally, the temporal average of $C_r$ and $|\psi_6|$ for all the measured parameters are shown [Fig. S3 (d-e)]. Both curves show a dependence on the symmetry of the base, being maximal when it is different from $\theta = 0°$ and $30°$, that is when it loses its crystalline symmetry. On the other hand, we again find an expected result when exploring the dependence of both parameters as a function of the injected energy; it is inversely proportional to $\Gamma$, i.e., the state becomes more fluid as its energy increases.

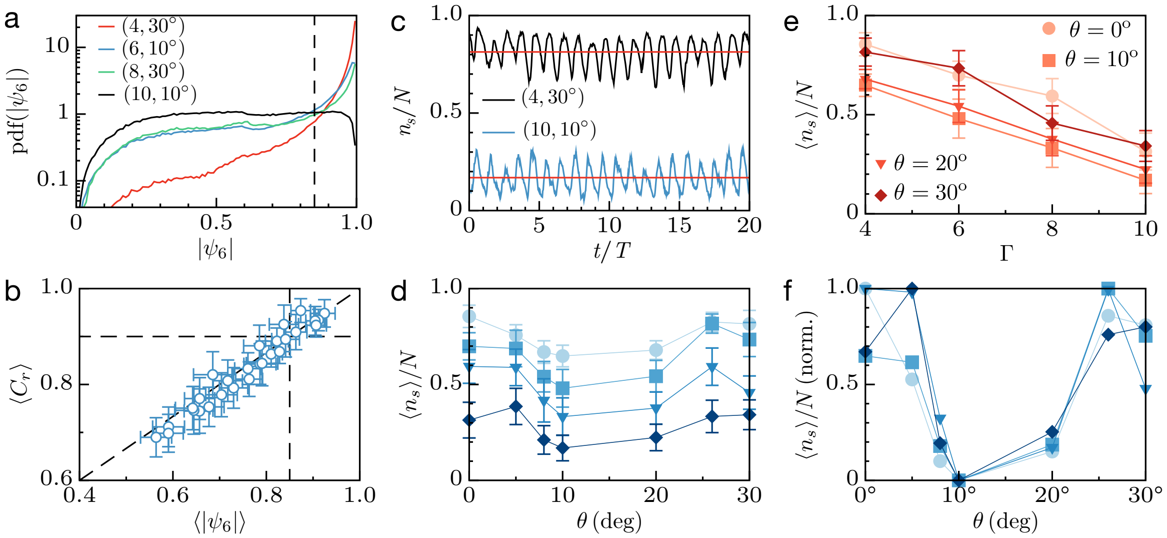


**Figure S4.** (a) Probability density function of the local order parameter for different $(\Gamma,\theta)$ configurations. Black dashed line represents the intersection point between the curves at $|\psi_6| = 0.85$. (b) Relative local compaction $\langle C_r\rangle$ vs order parameter $\langle|\psi_6|\rangle$. (c) Solid fraction as a function of time (black) $(\Gamma,\theta) = (4,30°)$ and (blue) $(\Gamma,\theta) = (10,10°)$. Red lines correspond to the respective average of each curve. (d) Solid fraction as a function of the angle. (e) Solid fraction as a function of the acceleration. (f) Normalized average of the solid fraction as a function of the angle. [The color legend used in (d) and (f) are the same as those shown in the figure S3 (e,d)].

## 3 Phase-detection and evolution

A local crystal structure can be identified using a criterion based on an order parameter (i.e., a well-defined symmetry such as a square or hexagons) or packing fraction (i.e., particles close together). Our results show that the order parameter $|\psi_6|$ is an excellent candidate to define this structure [Fig. S4 (a)]. It presents two well-separated regions, where $|\psi_6| \simeq 1$ gives a local maximum corresponding to particles ordered in a hexagonal array and a plateau around $|\psi_6| \simeq 0.5$ indicating particles in an amorphous state, i.e., with no apparent order. In between these two regions, we can estimate a local minimum at $|\psi_6^*| = 0.85$. Likewise, we found that the local relative compaction $C_r$ presents an almost linear correlation with the order parameter $\psi_6$ [Fig. S4 (b)]. We can extract that a similar criterion based on this parameter is satisfied for $C_r = 0.9$. The separation of the system states allows us to quantify the ratio of solid-to-fluid particles as $\langle n_S/N\rangle$, where $n_S$ is the number of solid particles as a function of time, and $N$ is the total number of particles in the sample. An example of the solid fraction in time is shown in Figure S4 where we observe that the configuration $(\Gamma,\theta) = (10,10°)$ is more fluidized than $(\Gamma,\theta) = (4,30°)$. In both cases, the solid fraction fluctuates at the frequency of the external oscillation. Since the percentage of solid particles is less than 1 ($n_s/N < 1$), the system always coexists between a crystal and an amorphous (fluid-like) state. Here, the most fluidized case is around 15% of particles in a solid-like state, whereas there is approximately 80% of such particles for the least fluidized case (see Figure 3 in the manuscript). The effects produced in the solid fraction by modifying the base geometry at constant acceleration are presented in Fig. S4 (d). Here, the solid fraction decreases when the acceleration increases and also at the angles where the symmetry of the hexagonal lattices is broken ($\theta \neq 0°, 30°$). On the other hand, Figure S4 (e) shows the average of the solid fraction as a function of acceleration. Each curve is at a constant angle. We observed that the solid fraction decreases monotonically when the acceleration increases, an expected result in a vibrated granular medium. Similarly, Figure S4 (f) shows the normalized time average of the solid fraction defined as $n_s(\text{norm.}) = (n_S - \min(n_S))/(\max(n_S) - \min(n_S))$ as a function of the angle. Here, it is more evident that the solid fraction decreases when the system has an angle that breaks the symmetry of the hexagonal lattices.

## 4 Statistical analysis of the thermal fluctuations

From particle tracking we can obtain the positions and velocities of all particles at each instant. This information is then used to study the statistical properties of the system. From the two directions of motion $(x,y)$ we can defined the velocity fluctuations of the particle $j$ in the $x$ (horizontal) or $y$ (vertical) directions as $\tilde{v}_{x,y}(j,t) = v_{x,y}(j,t) - \bar{v}_{x,y}(t)$. Where $\bar{v}$ denotes the ensemble average, over all particles at a given instant: it thus corresponds to the in-plane component of the instantaneous velocity of the

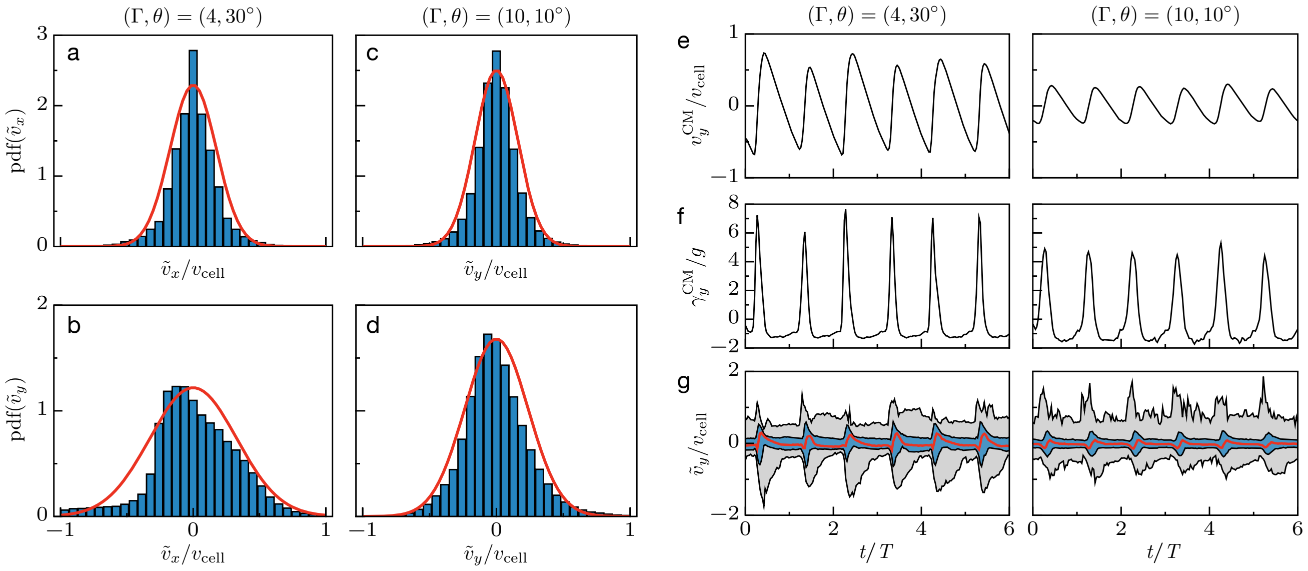


**Figure S5.** Probability density function of the (a,c) horizontal $\tilde{v}_x/v_{\mathrm{cell}}$ and (b-d) vertical $\tilde{v}_y/v_{\mathrm{cell}}$ velocity fluctuations for $(\Gamma,\theta)=(4,30°)$ and $(\Gamma,\theta)=(10,10°)$ respectively. The red lines correspond to the normal distribution fit. (e) Vertical velocity of the center of mass normalized by the velocity of the cell as a function of time. (f) Vertical acceleration of the center of mass normalized by gravity as a function of time. (g) Boxplot of the relative vertical velocity for (left) $(\Gamma,\theta)=(4,30°)$ and (right) $(\Gamma,\theta)=(10,10°)$. The red lines are the median; the blue filler is the range between the first and third quartile ($Q_1-Q_3$) and the grey filler is the range of maxima to minima velocities.

center of mass of the monodisperse sample, $\bar{v}_{x,y}(t)=v_{x,y}^{\mathrm{CM}}(t)$. The velocity of the center of mass is defined as,

$$v_{x,y}^{\mathrm{CM}}=\frac{1}{N}\sum_{j=1}^{N}v_{x,y}^{j}(j,t), \tag{S1}$$

where $N$ is the total number of particles and $v_{x,y}^{j}$ is the horizontal/vertical component of the velocity of the $j$-th particle. All the particles have the same mass. Figure S5 (a-d) shows both components of the probability density function of the velocity fluctuation normalized by the velocity amplitude of the cell $v_{\mathrm{cell}}=\Gamma g/\omega$. Figure S5 (e) shows the vertical velocity of the center of mass for $(\Gamma,\theta)=(4,30°)$ and $(\Gamma,\theta)=(10,10°)$. Two things are noteworthy. On the one hand, the motion of the synchronized particle cluster, i.e., when the particles are in a solid phase $(\Gamma,\theta)=(4,30°)$, has a larger amplitude of motion than in the fluid case $(\Gamma,\theta)=(10,10°)$. On the other hand, the shape of the signal (sawtooth wave) reflects the impact of the setup base with the set of grains, increasing its velocity drastically at each impact and decreasing linearly after it. Also, the center of mass velocity oscillates with the same frequency as the cell. Additionally, the dimensionless vertical acceleration of the center of mass, $\gamma_y^{\mathrm{CM}}/g$, describes a dynamic similar to that of the velocity with peaks marked with the oscillation period [Fig. S5 (f)]. The amplitude (width) of the signal are more pronounced (thin) when the system is in its solid-like state $(\Gamma,\theta)=(4,30°)$, than in the liquid case $(\Gamma,\theta)=(10,10°)$, where it presents lower intensity and wider peaks. The negative values of the acceleration correspond to the instant when the grains are in free fall. Finally, the evolution in time of the velocity distribution are presented in Figure S5 (g). The median (red lines) deviates from zero when the cell collides with the particles. In the same way, one can see that the range of quartiles (blue filler) and the range between maxima and minima (gray filler) increase when the granular medium's collisions on the cell happen.

## 5 Video examples

Finally, we provide four Supplementary Video files with representative values of $(\Gamma,\theta)$ showing the evolution of the particle velocity field, the topological defects (dislocations and disclinations), and the definition of solid and fluid particles (see the snapshots of these animations in Fig. S6).

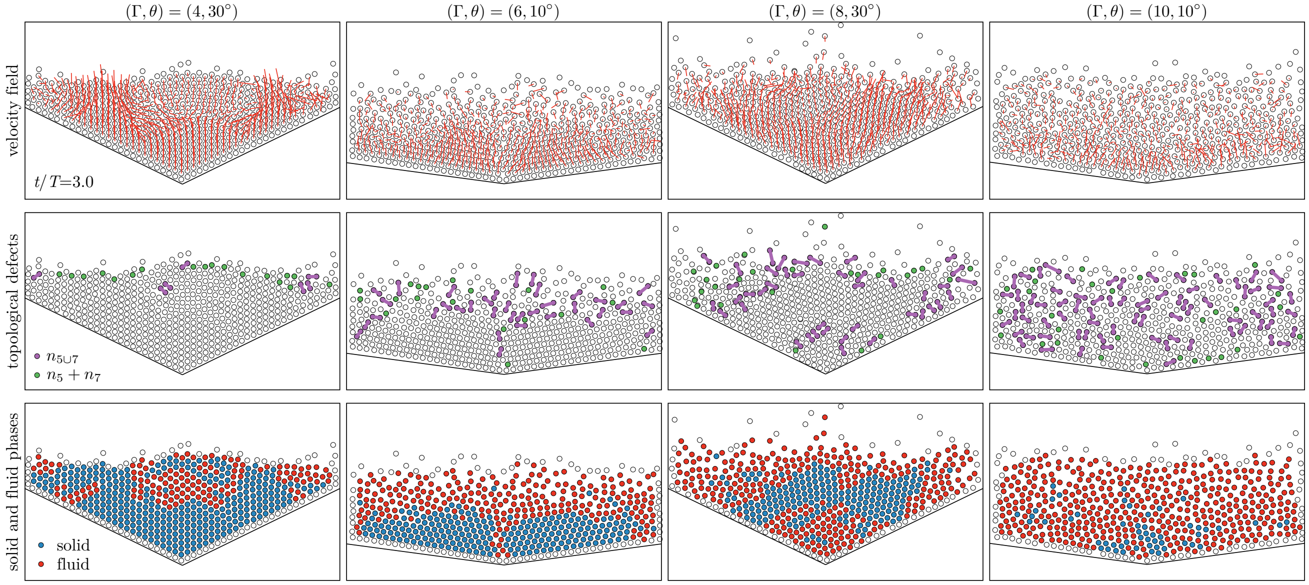


**Figure S6.** Snapshots at $t/T = 3$ for all the quantities measure in the experiment. (upper row) velocity field represented by the red arrows, (middle row) topological defects (dislocation $n_{5\cup7}$ and disclination $n_5$+$n_7$) and (lower row) solid and fluid phases.

## 6 Dynamic Lindemann parameter across phase transition

The crystalline solid-like phase differs from the hexatic and liquid-like phases by the mobility of the particles: they are caged by neighbors in the former case, whereas they can drift and even swap their positions in the two later cases[2,3]. Hence, the relative displacement $\vec{r}_{ij}(t) = \Delta\vec{u}_i(t) - \Delta\vec{u}_j(t)$ between a particle $i$ and a nearest neighbor $j$ plateaus at long time in the solid phase and diverges otherwise, being $\vec{u}_i(t)$ the position of the particle $i$ and $\Delta\vec{u}_i(t) = \vec{u}_i(t) - \vec{u}_i(0)$ its displacement. The dynamic Lindemann parameter[4] reflects these features, as it measures the root mean square distance traveled by a particle relatively to nearest neighbors,

$$L_d^2(t) = \langle|\vec{r}_{ij}(t)|^2\rangle/2a^2, \tag{S2}$$

where $a$ is the lattice constant, set equal to the diameter of the particles $a = d = 2$ mm, and where $\langle\dots\rangle$ denotes the instantaneous average over all nearest neighbors. Analyzing the trend of $L_d$ proved to be a reliable indicator to unveil transitions involving a solid-like phase[2–4]. In this perspective, the Fig. S7(a) reveals that none of the configurations $(\Gamma,\theta)$ we probed tends to a plateau. Our system is never observed in a pure solid-like crystal state, coherently with the fact that it always contains disclinations and dislocations, see the Fig. S1. In agreement with the conclusions presented in the manuscript, this suggests the coexistence of a liquid-like phase at any probed $kT$ and a phase transition at vanishing temperature, $kT = 0$, arising from the geometrical asymmetry of the container during the sample preparation. The dependence of $L_d$ on the geometry of the container is established in the Fig. S7(b), consistently with the trends of all the features presented in the manuscript (amount of topological defects, order parameter, compacity, solid fraction, temperature...): the asymmetry between the container and the lattice boosts the mobility of the particles in the intermediate range of V-shape angles $\theta$ at all amplitudes $\Gamma$. Alternatively, the Fig. S7(c) shows the same dataset, $L_d(t = 10T)$, but as a function of temperature $kT$. The former rises monotonically with the latter: as expected, increasing the thermal agitation enhances the mobility of the particles.

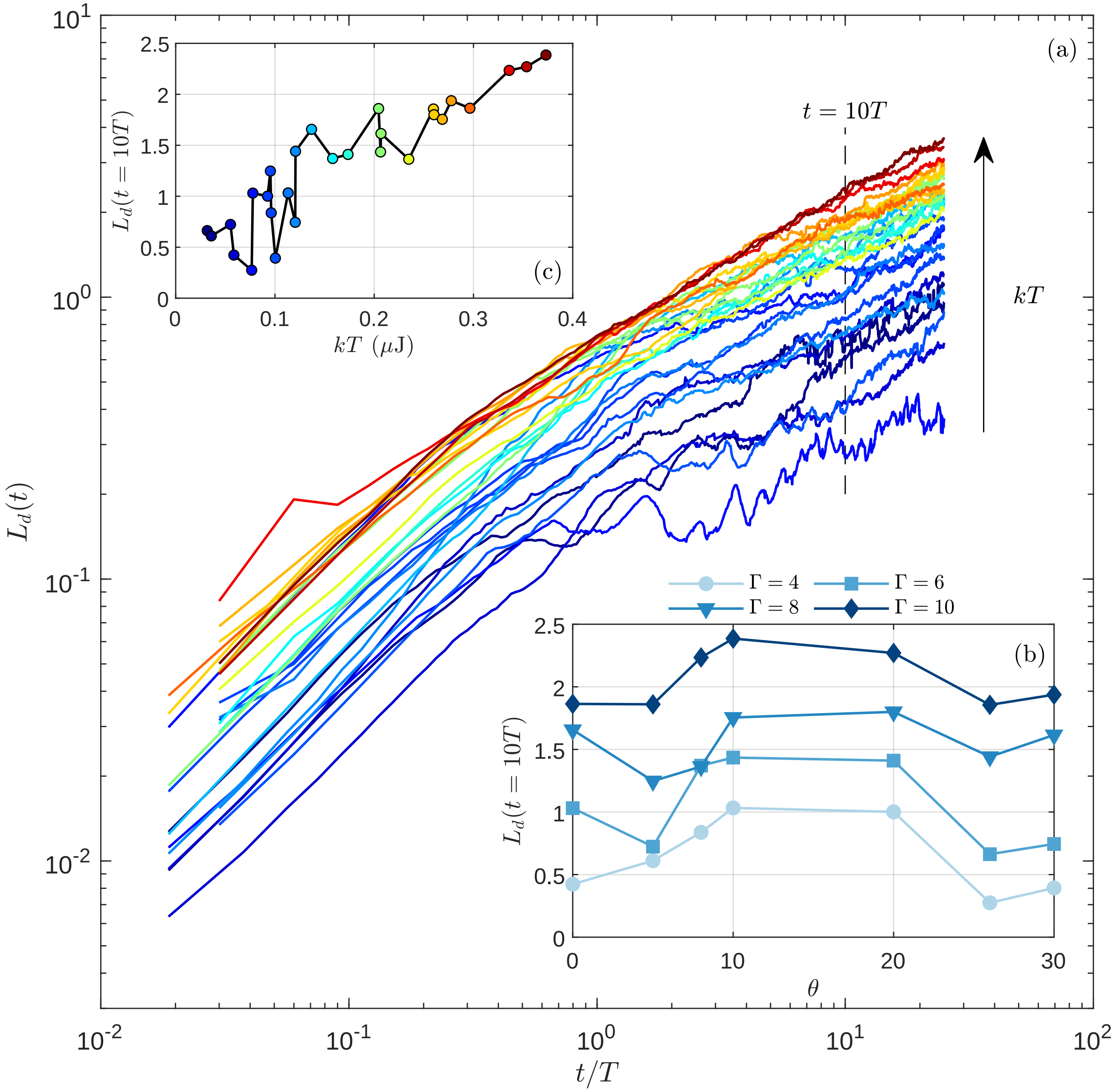


**Figure S7.** Dynamic Lindemann parameter (a) versus time $t$ for different temperatures $kT$, (b) versus $kT$ at $t = 10T$, and (c) versus angle $\theta$ for different amplitude $\Gamma$ at $t = 10T$.

## References


**1.** Shen, H., Tong, H., Tan, P. & Xu, L. A universal state and its relaxation mechanisms of long-range interacting polygons. *Nat. Commun.* **10**, 1737, DOI: 10.1038/s41467-019-09795-6 (2019).

**2.** Han, Y., Ha, N. Y., Alsayed, A. M. & Yodh, A. G. Melting of two-dimensional tunable-diameter colloidal crystals. *Phys. Rev. E* **77**, 041406, DOI: 10.1103/physreve.77.041406 (2008).

**3.** Sun, X., Li, Y., Ma, Y. & Zhang, Z. Direct observation of melting in a two-dimensional driven granular system. *Sci. Reports* **6**, 24056, DOI: 10.1038/srep24056 (2016).

**4.** Zahn, K. & Maret, G. Dynamic criteria for melting in two dimensions. *Phys. Rev. Lett.* **85**, 3656, DOI: 10.1103/PhysRevLett.85.3656 (2000).
